# Osteoporosis in adjacent cervical segments exacerbates disc herniation

**DOI:** 10.1038/s41598-025-06554-0

**Published:** 2025-07-02

**Authors:** Beiyang Wang, Yang Liu, Zhiqiang Wang, Chenguang Niu, Jian Tang, Lin Sun

**Affiliations:** 1 Third Hospital of Shanxi Medical University, Shanxi Bethune Hospital, Shanxi Academy of Medical Sciences, Tongji Shanxi Hospital, Taiyuan, 030032 China; 2 College of Mechanical and Vehicle Engineering, Taiy uan University of Technology, Taiyuan, People’s Republic of China; 3 Key Laboratory of Advanced Transducers and Intelligent Control System, Ministry of Education, Taiyuan University of Technology, Taiyuan, 030032 China

**Keywords:** Disc herniation, Cervical spine, Osteoporosis, Clinical follow-up, Biomechanics, Computational models, Clinical trial design, Endocrinology, Pathogenesis, Risk factors

## Abstract

Current studies into the relationship between osteoporosis and cervical disc herniation (CDH) remain inconclusive, primarily due to the scarcity of large-sample clinical follow-ups and biomechanical evidence. This retrospective, single-center study analyzed a consecutive series of 933 patients. We compared clinical parameters including age, gender, BMI, smoking history, alcohol consumption, steroid use history, thyroid dysfunction, diabetes history, and C5-C6 Hounsfield Unit (HU) values between groups with and without C5 disc herniation. Use multivariable-adjusted logistic regression to analyze the relationship between C5 and C6 vertebral body HU values and CDH. A finite element model was utilized to assess the impact of osteoporosis on cervical segments. In the Logistic regression analysis, after adjusting for confounding variables, the results showed that decreased HU values in the C5 (OR: 0.951, 95% CI: 0.911–0.992, *P* = 0.02) and C6 (OR: 0.923, 95% CI: 0.884–0.964, *P* < 0.001) segments were independent influencing factors for intervertebral disc herniation at the C5 level of the cervical spine. In addition, finite element analysis showed that articular cartilage stress decreased in the reduced bone density group, whereas disc stress and displacement increased. Osteoporosis of adjacent cervical segments increases the risk of disc herniation.

**Supplementary Information:**

The online version contains supplementary material available at 10.1038/s41598-025-06554-0.

## Introduction

CDH is a common spinal condition resulting from degenerative changes in the cervical disc and external factors that cause the fibrous ring to rupture, leading to a protrusion or prolapse of the nucleus pulposus tissue^1^. This condition compresses surrounding structures such as the spinal cord and nerve roots, resulting in a variety of clinical symptoms. However, the exact pathogenesis underlying CDH and degeneration remains unclear, posing a challenge for effective prevention^2^.

Osteoporosis, a prevalent bone disease, is characterized by reduced bone mass and microstructural damage to bone tissue. However, the relationship between CDH and adjacent vertebral osteoporosis remains unclear. Some studies suggest that lumbar osteoporosis correlates with increased intervertebral disc herniation^3,4^. In contrast, other research indicates osteoporosis may delay intervertebral disc degeneration, while anti-osteoporosis treatments could potentially accelerate it^5,6^. These discrepancies may stem from inconsistent bone density measurement protocols and limited large-sample clinical follow-up data.

The HU value is a quantitative parameter used to quantify tissue density in CT scan images. The HU value is an accurate assessment of bone density and has been widely used in osteoporosis screening and diagnosis^7^. In addition, HU values exhibit correlations with bone mechanical properties^8^, allowing accurate finite element biomechanical analysis.

This study retrospectively analyzed 933 patients and measured the HU values of C5 and C6 vertebrae to investigate the correlation between adjacent cervical vertebral density and disc herniation. And based on the HU values, finite element analysis was performed to explore the biomechanical mechanism between vertebral bone density and disc herniation. For the author’s knowledge, this is the first study to use large-scale clinical follow-up and biomechanical verification to investigate the relationship between cervical adjacent segment osteoporosis and CDH.

## Method

### Clinical follow-up data

This study was approved by the Ethics Committee of Shanxi Norman Bethune Hospital. Informed consent was obtained from all participants. This study included all patients hospitalized in our hospital from January 2014 to January 2024. All experiments were performed in accordance with relevant guidelines and regulations. The screening process for patient inclusion was conducted by two independent evaluators. In cases where the evaluators did not agree, a third evaluator was consulted to make the final decision.

Inclusion Criteria: 1. Case group: Patients presenting with neck pain, radicular symptoms, or spinal cord compression symptoms, clinically evaluated and diagnosed with C5 disc herniation by physicians, with confirmation through cervical MRI/CT imaging. 2. Control group: Imaging studies confirmed the absence of cervical C5 disc herniation, and no neurological deficits were identified.

Exclusion Criteria: Patients with spinal infections, tumors, trauma, radiation therapy, inflammatory diseases, or presence of internal fixation devices in the neck. Patient demographic data, including age, gender, BMI, smoking history, alcohol consumption, steroid use history, thyroid dysfunction, diabetes history. After applying the inclusion and exclusion criteria, 933 patients were ultimately included in the study.

**Fig. 1:**
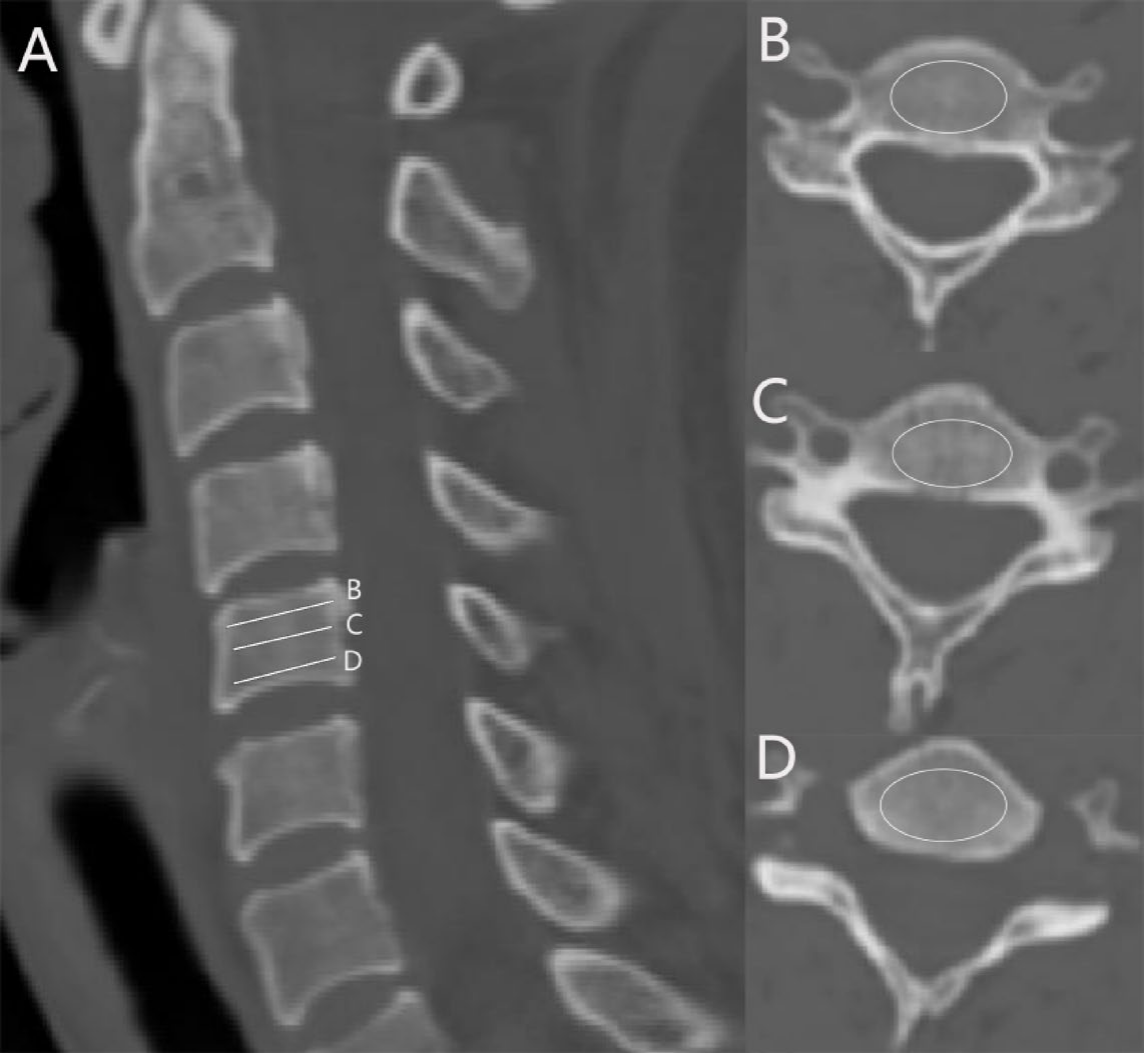
HU value measurement (**A**) Cervical sagittal position (**B**–**D**) Cervical horizontal ROI (region of interest) HU value measurement.

Method of HU measurement: CT imaging data were used to measure the HU value of the C5-C6 vertebral bodies according to a method previously described by our team^9^. The HU was determined by delineating an elliptical region of interest (ROI) that was centrally located within the cervical vertebrae, with the goal of including cancellous bone while excluding cortical bone margins, osteophyte formations, and bone sclerosis. The average HU value for each vertebral body was calculated based on the average HU values derived from three ROIs positioned horizontally across the cervical vertebrae (Fig. 1). All measurement data were independently evaluated twice by two spine physicians who were blinded to the clinical data. The average value between the two surgeons was taken as the final result.

Statistical Analysis: Statistical analysis was performed with SPSS 26.0 software. Continuous variables are presented as mean ± standard deviation and Median (interquartile range), while categorical variables are presented as frequencies. Independent samples t-tests and Mann–Whitney U tests were used to compare continuous variables, and chi-squared tests were used to compare categorical variables, the Pearson Chi-square test was employed when the sample size was n ≥ 40 and the expected frequency in each cell was T ≥ 5. When the sample size was n ≥ 40 but some cells had expected frequencies between 1 and 5 (1 ≤ T < 5), the continuity-corrected Chi-square test was applied to account for the potential bias in small expected frequencies. Use multivariable-adjusted logistic regression to analyze the relationship between C5 and C6 vertebral body HU values and CDH. Model 1 is unadjusted, and Model 2 is adjusted for age, gender, BMI, smoking history, alcohol consumption, steroid use history, and thyroid dysfunction. The receiver operating characteristics curve (ROC) was used to evaluate the value of predicting CDH, and the area under the curve (AUC) was calculated. A significance level of *P* < 0.05 was considered statistically significant.

### Finite element analysis

**Fig. 2:**
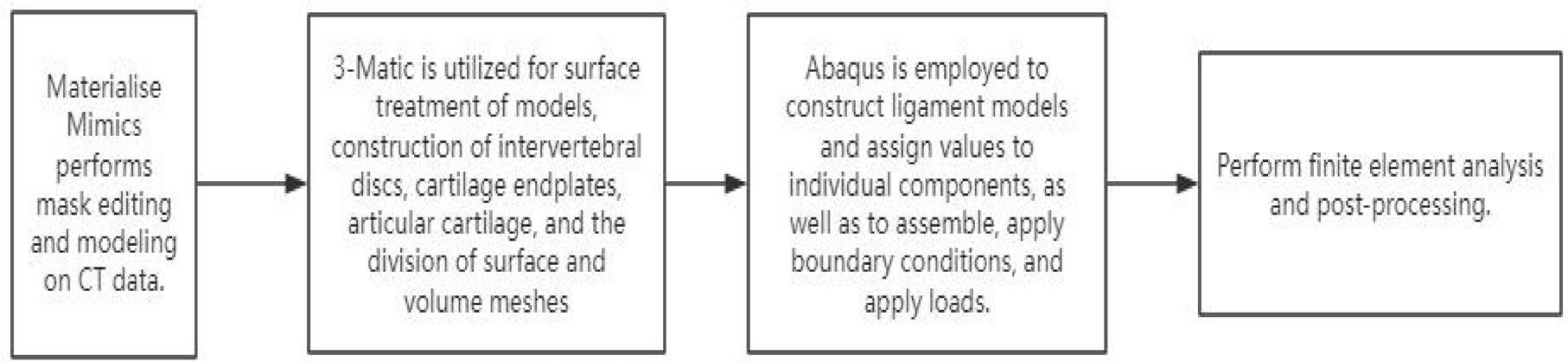
Diagram flowchart of the construction process of a finite element model.

**Fig. 3:**
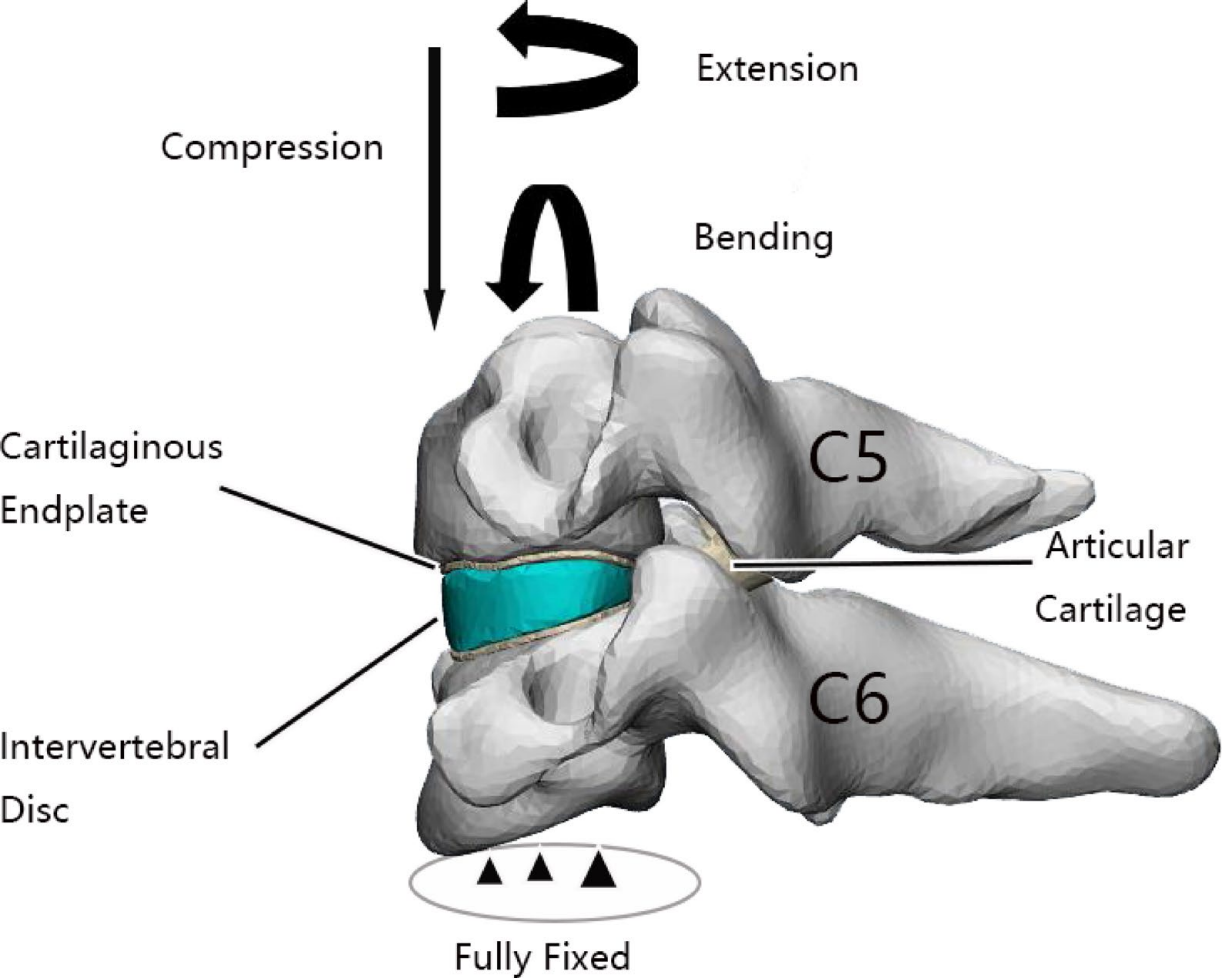
Finite element model schematic.

The flowchart for the finite element construction process has been outlined (Fig. 2). The finite element model construction is based on the CT data of a healthy volunteer’s cervical spine. The CT data is imported into Mimics21.0 to construct a C5-C6 mask. Subsequently, C5-C6 cortical bone, trabecular bone, endplate, articular cartilage, intervertebral disc and other structures are constructed in 3-Matic, and the model is optimized for surface and volume grids. The 3-matic software partitioned the model into tetrahedral mesh elements, with the finite element model comprising 80,096 mesh elements and 40,049 nodes. Subsequently, the structures of anterior longitudinal ligament(ALL), posterior longitudinal ligament(PLL), ligamentum flavum, intertransverse ligaments, supraspinous ligament, and capsular ligament were constructed in Abaqus, and the components and ligament materials were assigned values^10–12^ (Table 1). The component contact relationship is set as binding. The lower surface of C6 is completely fixed, while a downward force of 50 N is applied to the upper surface of C5 to simulate the weight of the head, with a bending moment of 1Nm. The load was applied at the center point of the upper surface, which was coupled with the upper surface of C5 (Fig. 3). Verify the effectiveness of the model by comparing the motion of the flexion, extension, bending, and rotation models with the literature^13^.

**Table 1:** Normal cervical spine material properties.

Component	E (MPa)	ν
Cortical bone^10^	12,000	0.3
Cancellous bone^14^	500	0.3
Nucleus^11^	1	0.49
Annulus Fibrosus^11^	3.4	0.4
Ligamentum Flavum^15^	1.5	0.3
Supraspinous Ligament^15^	1.5	0.3
ALL^15^	30	0.3
PLL^15^	20	0.3
Interspinous Ligament^15^	1.5	0.3
Capsular Ligament^15^	10	0.3
Cartilage Endplate^16^	23.8	0.3
Articular Cartilage^16^	23.8	0.3

Figure 3 was created using Mimics Research 21.0 (https://www.materialise.com/) and Adobe Photoshop 2023 (https://www.adobe.com/Photoshop).

We have constructed two distinct models, one representing the normal group and the other representing the osteoporosis group, with the elastic moduli of the trabecular bone set at 500 MPa and 300 MPa, respectively. We have measured the maximum stress values in the intervertebral discs and articular cartilage, as well as the maximum displacement of the intervertebral discs. Additionally, we calculated the difference between the osteoporosis group and the normal group, and used the percentage of the difference relative to the normal group to compare the differences between the two groups.

## Result

### Clinical follow-up data

**Fig. 4:**
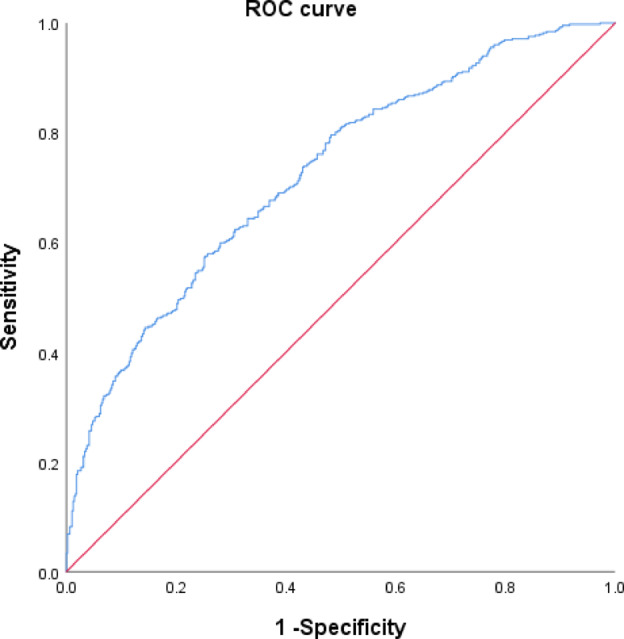
The ROC curve shows an area under the curve (AUC) of 0.725 (p < 0.001).

The study included 933 patients including 452 cases CDH of C5 and 481 cases Non-CDH of C5. Baseline characteristics including age, gender, BMI, smoking history, alcohol consumption, steroid use history, thyroid dysfunction, diabetes history were not significantly different between the two groups (p > 0.05). HU values of C5 and C6 vertebrae were significantly lower in the CDH group compared to the non-CDH group (p < 0.05) (Table 2). In multivariable analysis adjusted for confounders, multiple models of logistic regression showed that the HU values of C5 and C6 vertebrae were significantly associated with CDH(p < 0.05) (Tables 3, S1). The ROC curve shows an AUC of 0.725 (p < 0.01) (Fig. 4).

**Table 2:** Baseline characteristics.

Variables	CDH of C5	Non-CDH of C5	*P*
Age	55.47 ± 10.419	56.59 ± 11.007	0.11
Gender(male/female)	254/198	264/217	0.688
BMI	24.49(5.31)	24.97(4.79)	0.69
Smoking history (with/without)	149/303	173/308	0.335
Alcohol consumption (with/without)	86/366	88/393	0.774
Steroid use history (with/without)	4/448	1/480	0.334
Thyroid dysfunction (with/without)	7/445	1/480	0.062
Diabetes history (with/without)	62/390	59/422	0.51
The HU value of C5	343.33(106.5)	383.67(97.5)	< 0.001
The HU value of C6	302.5(111.36)	348(92.17)	< 0.001

**Table 3:** Association between C5/C6 segmental HU value and CDH: results of binary logistic regression analysis.

	OR [95% CI]	*P*
The HU value of C5	0.951[0.911, 0.992]	0.02
The HU value of C6	0.923[0.884, 0.964]	< 0.001
Age	0.963 [0.949, 0.977]	< 0.001
Gender(male/female)	0.874 [0.639, 1.196]	0.401
BMI	1.004 [0.962, 1.049]	0.410
Smoking history (with/without)	0.856 [0.6, 1.222]	0.392
Alcohol consumption (with/without)	0.957 [0.633, 1.447]	0.834
Steroid use history (with/without)	4.428 [0.47, 41.734]	0.194
Thyroid dysfunction (with/without)	16.349 [1.343, 199.055]	0.028
Diabetes history (with/without)	1.306 [0.849, 2.009]	0.224

### Finite element analysis

In this study, a finite element model of the C5-C6 segment was constructed. In our study, we measured the range of motion by averaging the angles between pre-force and post-force positions of three distinct points relative to the center of the lower surface. The model matches the measurements of Panjabi et al.^13^ in flexion, extension, bending, and rotation and has been validated for accuracy (Fig S1).

**Fig. 5:**
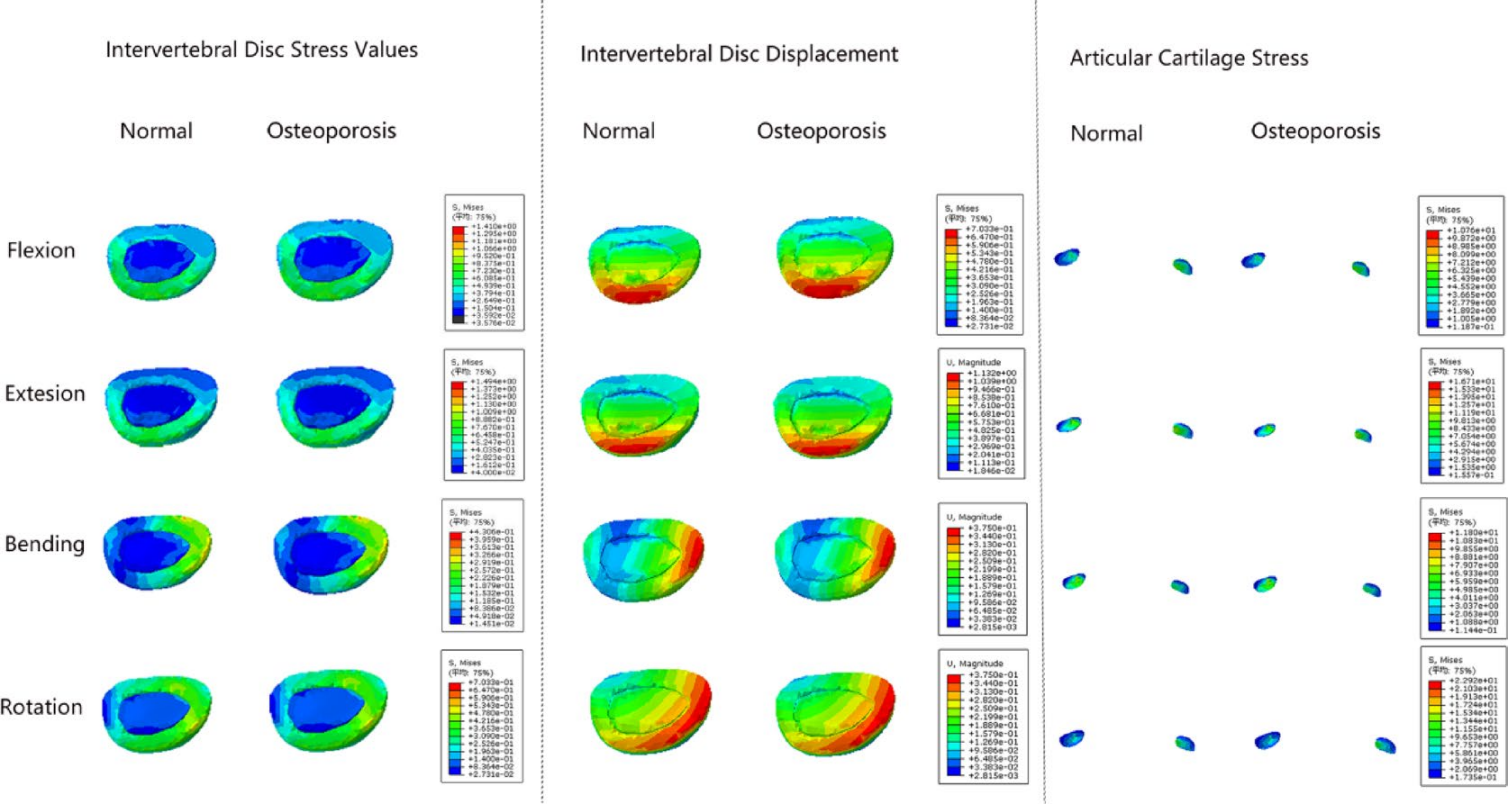
Stress and displacement cloud maps of intervertebral discs and articular cartilage in normal and osteoporotic groups.

Finite element analysis results indicate that the percentage of difference relative to the normal group in intervertebral disc stress for flexion, extension, bending, and rotation are 0.43%, 0.27%, 0.16%, and 0.028%, respectively. For displacement, the corresponding percentages for flexion, extension, bending, and rotation are 0.79%, 0.53%, 0.67%, and 0.19%. Regarding the stress in the articular cartilage, the percentages of difference relative to the normal group for flexion, extension, bending, and rotation are -0.09%, -0.12%, -0.17%, and -2.49%, respectively. Although the differences are minor, the trends consistently demonstrate that even mild osteoporosis can result in a decrease in stress on the articular cartilage and an increase in stress and displacement of the intervertebral discs during movement. Additionally, the stress distribution map of the intervertebral disc reveals that the stress is primarily concentrated in the annulus fibrosus rather than the nucleus pulposus. This suggests that when the intervertebral disc is subjected to increased forces, annular fibers are more prone to rupture (Fig. 5).

## Discussion

In this study, we measured the HU values of adjacent cervical segments, analyzed their correlation with disc herniation, and established a finite element model based on the HU values for biomechanical verification. The clinical follow-up results showed that the decrease in density of adjacent cervical segments was associated with CDH. Biomechanics shows that a decrease in bone density leads to a decrease in stress on articular cartilage, which in turn leads to disc displacement and increased stress.

CDH and osteoporosis are the most common diseases among middle-aged and elderly people^17^. Various treatments for osteoporosis have demonstrated significant efficacy in improving outcomes^18^. However, effective preventive measures for CDH remain limited. At present, there is still considerable controversy regarding the relationship between CDH and osteoporosis^19,20^. Therefore, investigating the correlation and causation between these conditions is critical to advancing CDH prevention strategies.

Through HU measurement and clinical follow-up, we observed a positive correlation between decreased density of adjacent cervical segments and disc herniation. Consistent with our research, Zhong et al.^21^ used Miro-CT to measure bone density in 12 rhesus monkeys and found that osteoporosis accelerated disc degeneration. A study of 105 postmenopausal women using quantitative Dixon and GRAPPA T2-mapping techniques also linked osteoporosis to disc degeneration^22^. In contrast, Zhang et al.^23^ found a negative association between osteoporosis and disc degeneration using dual-energy X-ray absorptiometry in 114 postmenopausal women. A study evaluating bone density using vertebral quality scoring in 130 patients with lumbar spinal stenosis and disc herniation found no association^24^. Perhaps due to different measurement methods and insufficient sample size. The HU value is a reliable method to reflect bone density and has been widely used in the diagnosis of osteoporosis. In this study, we used HU values to estimate bone density by measuring C5-C6 segment HU values in 933 patients with cervical spondylosis and conducted clinical follow-up to explore correlations with disc herniation.

Many studies have investigated the specific causal relationship between osteoporosis and disc herniation using various methods. Zhang et al.^25^ found that mice with reduced bone density had significantly fewer blood vessels in the endplate, the lack of blood supply reduced nutrition to the disc, contributing to disc degeneration. Xi et al.^26^, using diffusion tensor imaging to assess stress in the L4-L5 segment, suggested that osteoporosis worsens local disc biomechanics. Zhao et al.^27^, using Mendelian randomization analysis, confirmed the causal relationship between lower bone density and disc degeneration. However, there are some studies suggest that osteoporotic vertebrae may buffer disc loading and that anti-osteoporotic treatments may accelerate disc degeneration^5,6^. Some researchers have suggested that varying degrees of bone density reduction can lead to different effects on intervertebral disc herniation^20^. The controversy in these studies may be due to the lack of realistic simulations of the osteoporotic environment and accurate biomechanical analysis.

The results of finite element analysis indicate that as the bone density of adjacent cervical vertebrae decreases, the stress on the articular cartilage decreases, resulting in increased stress and displacement of the intervertebral disc. The direct contact and small contact surface between the articular cartilage of the upper and lower cervical vertebrae causes stress concentration^28,29^. With decreasing bone density, the articular cartilage initially experiences reduced stress, resulting in a seesaw-like effect, which leads to increased stress and displacement of the disc. In addition, our study found that stress in the annular fibrosus was significantly higher than in the nucleus pulposus. Therefore, in osteoporosis, the annulus fibrosus may rupture first, followed by protrusion of the nucleus pulposus.

For patients with low bone density, additional measures such as medication or lifestyle modifications may be necessary to enhance bone density, thereby reducing the likelihood of cervical disc herniation. This offers a novel perspective for the prevention of cervical disc herniation. By monitoring and improving bone density, it may be possible to slow down the process of disc degeneration and improve patient outcomes.

There are several limitations to this study. First, this study only included HU values of C5-C6 segments for research, as the sample size of other segments with intervertebral disc herniation was relatively small. Secondly, our finite element model currently focuses on constructing skeletal and ligamentous structures to study the relationship between osteoporosis and intervertebral discs. We aim to further explore and refine the model in our future research. Finally, in addition to the HU value, there are other methods for detecting bone density, such as C-VBQ. More detection methods will be further applied in the next step of future research.

## Conclusion

In summary, clinical follow-ups and biomechanical analyses indicate that decreased bone density in adjacent cervical segments increases the risk of disc herniation.

## Electronic supplementary material

Below is the link to the electronic supplementary material.

Supplementary Material 1

Supplementary Material 2

Supplementary Material 3

## Data Availability

Data is provided within the manuscript or supplementary information files.

## Abbreviations

CDH: Cervical disc herniation
HU: Hounsfield unit
