# Supplementary material for: Osteoporosis in adjacent cervical segments exacerbates disc herniation
Source: Sci Rep. 2025 Jul 2;15:22901. doi: 10.1038/s41598-025-06554-0 (PMC12214985; doi:10.1038/s41598-025-06554-0)
Supplement: Supplementary file 3 — Supplementary Material 3 [file 41598_2025_6554_MOESM3_ESM.docx]

**Figure S1 Validity verification of finite element model for C5-C6 segments**

|  | **Model1 (OR [95% CI])** | ***P*** |
| --- | --- | --- |
| The HU value of C5 | 0.958[0.920, 0.999] | 0.043 |
| The HU value of C6 | 0.934[0.896, 0.974] | 0.001 |

|  | **Model2(OR [95% CI])** | ***P*** |
| --- | --- | --- |
| The HU value of C5 | 0.951[0.911, 0.992] | **0.02** |
| The HU value of C6 | 0.923[0.884, 0.964] | **<0.001** |
| Age | 0.963 [0.949 , 0.977] | **<0.001** |
| Gender(male/female) | 0.874 [0.639 , 1.196] | 0.401 |
| BMI | 1.004 [0.962 , 1.049] | 0.410 |
| Smoking history  (with / without) | 0.856 [0.6 , 1.222] | 0.392 |
| Alcohol consumption  (with / without) | 0.957 [0.633 , 1.447] | 0.834 |
| Steroid use history  (with / without) | 4.428 [0.47 , 41.734] | 0.194 |
| Thyroid dysfunction  (with / without) | 16.349 [1.343 , 199.055] | **0.028** |
| Diabetes history  (with / without) | 1.306 [0.849 , 2.009] | 0.224 |

**TableS1 Association Between C5/C6 Segmental HU Value and CDH: Results of Binary Logistic Regression Analysis**

**Note. Model 1: No covariates were adjusted. Model 2: Adjusted for age, gender, BMI, smoking history, alcohol consumption, steroid use history and thyroid dysfunction. OR = odds ratio; CI = confidence interval; BMI = body mass index; HU = Hounsfield Unit; CDH = Cervical Disc Herniation.**
